# A Hybrid Qualitative–Quantitative FMEA Model for Risk Management in Clinical Laboratory Automation: A Case Study Integrating ISO 15189:2022

**DOI:** 10.1002/jcla.70194

**Published:** 2026-03-14

**Authors:** Mengqi Wei, Mingyang Li, Yu Lin, Bowen Li, Hao Xue, Yong Xia

**Affiliations:** ^1^ Department of Clinical Laboratory Peking University Shenzhen Hospital Shenzhen China

**Keywords:** failure mode and effects analysis, ISO 15189:2022, risk management, total laboratory automation

## Abstract

**Background:**

Total laboratory automation (TLA) system greatly enhances testing efficiency and accuracy, yet potential process‐related risks may cause inaccurate results or delayed reports. Based on the ISO 15189:2022 standard, this study applied failure mode and effects analysis (FMEA) to systematically assess risks across three TLA systems at Peking University Shenzhen Hospital.

**Methods:**

The Roche, Abbott, and Beckman TLA systems were evaluated across the pre‐analytical, analytical, and post‐analytical phases. Failure modes were scored for severity (*S*), occurrence (*O*) and detectability (*D*) to calculate the risk priority number (RPN = *S* × *O* × *D*). To improve the objectivity of occurrence scoring, qualitative questionnaires were integrated with quantitative data from laboratory information system, equipment logs and quality control records. Targeted interventions were implemented for high‐risk nodes, and their effectiveness was subsequently reevaluated.

**Results:**

Several common high‐risk nodes were identified across the three systems, including sample transport delay, system malfunction, and reagent failures. Targeted interventions, such as pneumatic transport systems, data visualization dashboards, and intelligent reagent management platforms, were applied accordingly. Beckman's system‐specific quality control risks were effectively mitigated through Patient‐Based Real‐Time Quality Control and reaction curve monitoring. Result‐clinical consistency was improved in the Abbott and Beckman systems via retesting and multi‐rule verification. RPN values of critical failure modes were reduced to medium or low risk.

**Conclusion:**

By integrating ISO 15189:2022 with the FMEA approach, a standardized risk assessment model for TLA systems was established. The qualitative–quantitative integrated scoring system enhanced the objectivity of risk evaluation, while targeted intelligent interventions substantially improved the quality and safety of laboratory automation.

## Graphical abstract

This study integrates ISO 15189:2022 requirements with failure mode and effects analysis (FMEA) to establish a comprehensive risk management framework for multibrand total laboratory automation (TLA) systems. By combining quantitative operational data with expert evaluation, an objective and reproducible occurrence scoring strategy was developed. Targeted intelligent interventions effectively mitigated high‐risk failure nodes, leading to substantial reductions in risk priority numbers and significant improvements in analytical accuracy, operational reliability, and patient safety.

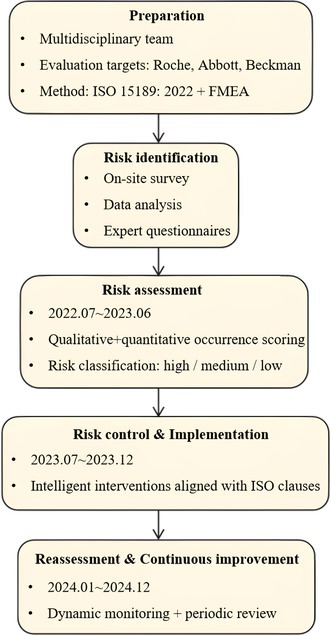

## 1 Introduction

With the continuous evolution of medical diagnostic models, medical laboratories have assumed an increasingly prominent role in disease screening, therapeutic monitoring and public health management [1, 2, 3, 4]. The growing demand for testing has imposed higher requirements on analytical efficiency and quality. In this context, total laboratory automation (TLA) systems have been widely adopted as key instruments to enhance testing capacity and ensure data accuracy [5, 6, 7].

Compared with traditional laboratory models, TLA systems significantly improve testing efficiency and standardization but also introduce new challenges in quality control and systemic risk management. First, hardware failures in any module—such as the track system, centrifugal unit or analytical module—can trigger cascading process disruptions. Second, interruptions in middleware, laboratory information system (LIS), or system interfaces may lead to widespread data‐chain failures. Third, the high‐throughput and high‐volume processing inherent to TLA can amplify the impact of isolated errors. Last, the unattended operation of fully automated pipelines may create monitoring blind spots, whereby latent issues are often detected only at later stages of testing. Consequently, conventional quality management models that rely on manual review and static regulations struggle to identify and mitigate such dynamic, process‐driven risks in real time [8].

The ISO 15189:2022 standard explicitly requires medical laboratories to implement systematic risk identification and comprehensive quality control throughout the entire testing process, thereby ensuring data traceability, timeliness, and reliability in compliance with clinical and regulatory expectations [9]. This standard encompasses the full chain of laboratory operations, including equipment, information systems, and analytical processes, emphasizing proactive risk surveillance and continuous quality improvement. Within fully automated laboratory environments, ISO 15189:2022 provides both a theoretical foundation and an operational guideline for constructing closed‐loop risk management systems that minimize cascading failures, strengthen data security, and enhance clinical applicability.

Failure mode and effects analysis (FMEA) is a well‐established risk management tool in the medical field. By systematically identifying potential failure modes and quantifying their severity, occurrence, and detectability, FMEA calculates a risk priority number (RPN) to support hierarchical intervention and prioritized control [10, 11]. Although FMEA has been applied in clinical laboratories, most studies have focused on individual devices or isolated processes, lacking comparative analysis across multibrand and full‐process automation systems. Furthermore, conventional FMEA scoring mechanisms are often limited by subjectivity, underutilizing the potential of data‐driven assessment supported by laboratory informatics [12].

In this study, conducted at Peking University Shenzhen Hospital, three TLA systems (Roche, Abbott, and Beckman) were systematically evaluated using the FMEA approach under the ISO 15189:2022 framework. A hybrid qualitative–quantitative scoring model was developed to enhance the objectivity, reproducibility, and scientific rigor of risk assessment. Through cross‐system comparison, system‐specific and shared risk patterns were identified, and differentiated control strategies were proposed, aiming to establish a practical and generalizable model for risk management and continuous quality improvement in TLA.

## 2 Subjects and Methods

### 2.1 Subjects

This study was conducted at Peking University Shenzhen Hospital, a representative tertiary general hospital in Shenzhen. The hospital's medical laboratory is equipped with three biochemical and immunological automation systems—Roche (CCM), Abbott (GLP), and Beckman (DxA 5000), which together undertake routine biochemical and immunological testing (Table 1). To ensure comprehensive quality assurance, the risk management framework established in this study encompassed the pre‐analytical, analytical, and post‐analytical phases, thereby achieving systematic control across the entire workflow from sample collection to result reporting.

**TABLE 1:** The characteristics of three biochemical and immunological automation systems.

	Roche (CCM)	Abbott (GLP)	Beckman (DxA 5000)
Component modules	Specimen loading module × 1	Transport module × 1	Transport module × 1
	Centrifuge module × 1	Input and output module × 1	Input module × 1
	Classification and cup‐distribution module × 1	Opening lid module × 1	Centrifuge module × 1
		Centrifuge module × 2	Output module × 1
		Buffer module × 1	Rack builder unit × 2
		Storage device × 1	Storage device × 1
Analyzer	e801 × 2 (Immunoassay)	Alinity I × 3 (Immunoassay)	DxI 800 × 4 (Immunoassay)
	e602 × 1 (Immunoassay)		AU 5800 × 2 (Biochemistry)
Test items	30 items	26 items	104 items (31 open reagents)
	TORCH, infection, bone metabolism, etc.	Tumor markers, preoperative routine items, etc.	Biochemistry (liver, kidney, myocardial enzymes, electrolytes, blood lipids, etc.) Immunoassay (sex hormones, thyroid, etc.)
System features	1. Photography function quickly determines serum quality	1. Static unpowered track	1. Fast sample processing
	2. Fast detection speed, with 93% items completed within 18 min	2. Compact modules save space	2. Low track failure rate
	3. Equipped with a classification and cup‐distribution module		

### 2.2 Methods

#### 2.2.1 Risk Identification

**FIGURE 1:**
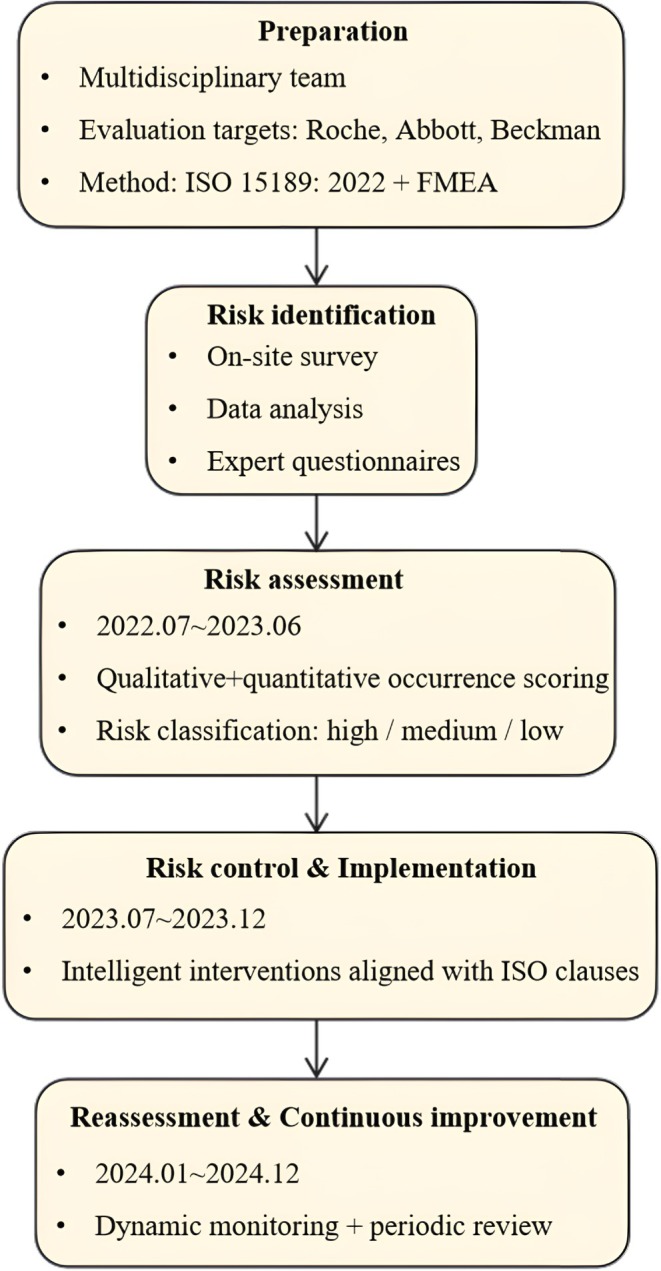
The flow diagram of risk management.

As shown in Figure 1, the overall workflow of the risk management process was illustrated based on the ISO 15189:2022 framework and the FMEA methodology. A multidisciplinary risk assessment team was established, consisting of five senior laboratory technologists, three equipment engineers, two quality management experts, and one information technology specialist. Under the ISO 15189:2022 framework, potential risk points were systematically reviewed and categorized according to relevant clauses, including 5.6 (risk management), 6.3 (facilities and environmental conditions), 6.4 (equipment), and 6.6 (reagents and consumables), to ensure compliance with standardized quality management requirements. Through on‐site investigations, analysis of LIS data, instrument operation logs, quality control software records, and expert panel discussions, potential risks were comprehensively identified across the pre‐analytical, analytical, and post‐analytical phases. These risks covered personnel‐related operational errors, equipment malfunctions, information system failures, and environmental factors.

#### 2.2.2 Risk Assessment

Quality index from July 1, 2022, to June 30, 2023, was collected. All identified failure modes were scored for severity (*S*), occurrence (*O*), and detectability (*D*) using a five‐point scale (criteria shown in Table 2). The risk priority number (RPN) was calculated as RPN = *S* × *O* × *D*. Risk levels were classified as follows: RPN ≥ 50, high risk (requiring immediate corrective action); 25 ≤ RPN < 50, medium risk (requiring enhanced monitoring and management); and RPN < 25, low risk (manageable under routine procedures).

**TABLE 2:** The RPN scoring criteria.

Risk score	Severity (*S*)	Occurrence (*O*)		Detection (*D*)
		Qualitative	Quantitative	
1	A. Increased workload or cost	Once in more than 5 years	0.2 ≤ Annualized occurrence < 1	A. Easily identified after simple training
				B. Automatically identified and alerted in real time
2	B. Delay in laboratory results	Once a year	1 ≤ Annualized occurrence < 4	C. Identified after routine training
	C. Delay in routine diagnosis or treatment			
3	D. Repeated sample collection procedures	Once a quarter	4 ≤ Annualized occurrence < 12	D. Identified after multiple training combined with professional skills
	E. Incorrect laboratory results			E. Identified through specific instruments or reagents
	F. Delay in important emergency diagnosis or treatment			F. Identified through manual analysis with LIS data
4	G. Misdiagnosis or inappropriate treatment	Once a month	12 ≤ Annualized occurrence < 52	G. Identified only after extensive training and strong professional skills
	H. Delay in critical emergency diagnosis or treatment			
5	I. Life‐threatening risk to the patient	Once a week	Annualized occurrence ≥ 52	H. Difficult to detect using current methods

To improve the comprehensiveness and applicability of occurrence scoring, both quantitative and qualitative approaches were integrated. Quantitative data including quality index from LIS, equipment operation logs, and quality control software records were used to calculate the actual frequency of risk events. For risks difficult to quantify, questionnaire surveys and expert panel evaluations were used to provide professional judgments of occurrence likelihood. Both data types were then mapped onto a unified scoring scale to ensure comparability and consistency.

#### 2.2.3 Rationale for Targeted Interventions

Targeted intervention measures were selected based on RPN ranking, system‐specific vulnerability, and compliance with ISO 15189:2022 requirements. Failure modes classified as high risk were prioritized for immediate corrective action. For failure modes shared across different automation platforms, standardized control strategies were adopted to ensure consistency and comparability. In contrast, system‐specific risks were addressed using differentiated, platform‐adapted interventions according to reagent configuration, workflow characteristics, and information system architectures. All interventions were designed with feasibility and sustainability in routine laboratory practice in mind, enabling long‐term risk control and continuous quality improvement.

#### 2.2.4 Risk Control

Targeted control measures were implemented for each high‐risk node identified in the assessment, with strategies tailored to the operational characteristics of the three biochemical and immunological automation systems. In the pre‐analytical phase, interventions focused on improving sample transport efficiency. During the analytical phase, system stability, quality control surveillance, and contingency mechanisms were strengthened. In the post‐analytical phase, verification procedures were optimized to minimize discrepancies between laboratory results and clinical features.

#### 2.2.5 Risk Reassessment

Six months after implementation of the intervention measures, a one‐year follow‐up evaluation was conducted from January 1 to December 31, 2024. Using the FMEA approach, post‐intervention risk scoring was performed to verify the effectiveness of the implemented control measures and to evaluate changes in the overall risk profile of the automated systems.

## 3 Results

### 3.1 Comprehensive Risk Assessment of Biochemical and Immunological Automated Systems

Before intervention, the three automation systems demonstrated both shared and system‐specific risk profiles. Sample transport delay represented a common high‐risk node across all platforms, whereas Beckman exhibited a distinctive vulnerability in quality control‐related failure modes, primarily due to its use of open reagents. In contrast, Roche and Abbott systems, which rely on closed reagent configurations, showed relatively lower analytical instability, with risks more concentrated in workflow coordination and system‐level malfunctions.

In the pre‐analytical phase, all three automated systems exhibited a common high‐risk node‐sample transport delay. The primary causes included prolonged transport intervals and insufficient personnel allocation, resulting in sample retention and compromised timeliness of subsequent testing (Table 3).

**TABLE 3:** Risk assessment of pre‐analytical phases in three biochemical and immunological automation systems.

Failure mode	Potential cause	Control measures	ISO 15189:2022 clause	Roche (annual samples: 121,212)				Abbott (annual samples: 490,925)				Beckman (annual samples: 829,645)			
				*S*	*O* (frequency, annual occurrence)	*D*	Risk level	*S*	*O* (frequency, annual occurrence)	*D*	Risk level	*S*	*O* (frequency, annual occurrence)	*D*	Risk level
Barcode error	Blurred/Incomplete barcode	1. Regular printer maintenance	7.2.3	1	5	1	Low	1	5	1	Low	1	5	1	Low
	Improper barcode pasting	2. Standardized barcode pasting			(0.31%, 376)				(0.31%, 1521)				(0.31%, 2572)		
		3. Barcode integrity detection at automation entry													
Mismatch between patient and tube information	Barcode attached to wrong tube	1. Double‐check protocol	7.2.3	4	2	4	Medium	4	2	4	Medium	4	3	3	Medium
		2. Compare with historical results			(0.0006%, 1)				(0.0006%, 3)				(0.0006%, 5)		
Incorrect tube type	Tube type not matching test requirements	1. Strengthen personnel training	7.2.4	3	2	3	Low	2	3	3	Low	3	4	3	Medium
		2. Automated sample type recognition system (R/B)			(0.0016%, 2)				(0.0016%, 8)				(0.0016%, 13)		
Abnormal sample condition	Hemolysis	1. Collection and transport training	7.2.4	3	4	3	Medium	2	5	4	Medium	3	5	3	Medium
	Lipemia	2. Serum index detection system (B)			(0.038%, 46)				(0.038%, 187)				(0.038%, 315)		
	Jaundice	3. Sample photography system (R)													
Insufficient sample volume	Poor collection technique or patient condition	1. Strengthen personnel training	7.2.4	3	5	3	Medium	2	5	3	Medium	3	5	2	Medium
		2. Automatic sample volume recognition system			(0.31%, 376)				(0.31%, 1521)				(0.31%, 2572)		
Improper sample preprocessing (human error)	Error in collection time	1. Strengthen personnel training	7.2.4	4	4	3	Medium	4	5	2	Medium	4	5	2	Medium
	Improper sample handling; Sample contamination	2. Review rules to automatically recognize abnormal samples (B)			(0.027%, 35)				(0.027%, 133)				(0.027%, 224)		
Sample transport delay	Long transport intervals	1. Shorten intervals	7.2.5	4	5	3	High	4	5	3	High	4	5	3	High
	Insufficient transport personnel	2. Optimize routes			(11.5%, 13,939)				(11.5%, 56,456)				(11.5%, 95,409)		
		3. Increase transport personnel													
Sample omission	Incomplete collection records	1. Strengthen personnel training	7.2	3	1	3	Low	5	1	4	Low	5	1	4	Low
	Unclear handover	2. Optimize handover			(0.0001%, 0.1)				(0.0001%, 0.5)				(0.0001%, 0.8)		
	LIS malfunction	3. LIS scanning registration													
		4. Timely receipt													

Abbreviations: A, Abbot; B, Beckman; R, Roche.

In the analytical phase, system‐specific differences were observed. The Beckman system demonstrated a unique high‐risk issue related to quality control failure, mainly attributed to the use of open reagents in certain biochemical assays, which was more susceptible to operational errors such as incorrect reagent loading. In contrast, the Roche and Abbott systems, which employ closed reagents, demonstrated lower quality control–related risks due to higher operational standardization. Despite these differences, all three systems shared common high risks during testing, including hardware or software malfunctions and reagent‐related failures, manifested as operational or data transmission interruptions and improperly stored or expired reagents (Table 4).

**TABLE 4:** Risk assessment of analytical phases in three biochemical and immunological automation systems.

Failure mode	Potential cause	Control measures	ISO 15189:2022 clause	Roche (annual samples: 121,212)				Abbott (annual samples: 490,925)				Beckman (annual samples: 829,645)			
				*S*	*O* (frequency, annual occurrence)	*D*	Risk level	*S*	*O* (frequency, annual occurrence)	*D*	Risk level	*S*	*O* (frequency, annual occurrence)	*D*	Risk level
Calibration failure	Untimely calibration	1. Automatic calibration reminders	6.5.2	3	3	3	Medium	3	3	2	Low	3	3	4	Medium
	Expired calibrators	2. Periodic check of calibrator validity			(Quarterly, 4)				(Quarterly, 4)				(Quarterly, 4)		
QC failure	Reagent misplacement	1. Standardized reagent placement (R/B)	7.3.7.2	4	4	3	Medium	4	5	2	Medium	4	5	4	High
	Expired reagents or QC	2. Periodic calibration			(0.42%, 30 items, 46)				(1.11%, 26 items, 105)				(2.11%, 104 items, 801)		
	Untimely calibration	3. Periodic check of reagent and QC validity													
Software failure	Software crash or freeze	1. Regular software updates and patch installation after validation	7.6	5	3	5	High	5	3	4	High	5	3	4	High
	Middleware or LIS/HIS interface error	2. Enhanced middleware/LIS/HIS interface stability			(Quarterly, 4)				(Quarterly, 4)				(Quarterly, 4)		
	Incorrect software configuration or update	3. Periodic check of software system													
Hardware failure	Instrument failure	1. Increased frequency of instrument maintenance	6.4	4	5	3	High	4	5	3	High	4	5	4	High
	Blocked sample track	2. Speed up engineer response			(Weekly, 52)				(Weekly, 52)				(Weekly, 52)		
	Module error														
Sample cross‐contamination	Incomplete cleaning of probe/tubing	1. Regular cleaning/replacement of probe/tubing	6.4.5	4	2	3	Low	4	3	3	Medium	4	3	4	Medium
		2. Enable reflex testing mode (auto‐retest for weak positives) (B).			(Once a year, 1)				(Quarterly, 4)				(Quarterly, 4)		
Reagent expiration	Improper storage conditions	1. Strictly follow storage guidelines	6.6	4	3	5	High	4	3	5	High	5	3	5	High
	Expired reagents	2. Periodical check of reagent validity			(Quarterly, 4)				(Quarterly, 4)				(Quarterly, 4)		
Temperature or humidity anomaly	HVAC failure	1. Set up temperature‐and‐humidity–monitoring and alarm system	6.3	3	2	3	Low	3	2	3	Low	4	2	3	Low
	Environmental monitoring failure	2. Regular maintenance of air conditioning and dehumidifier			(Once a year, 1)				(Once a year, 1)				(Once a year, 1)		

Abbreviations: HIS, hospital information system; HVAC, heating, ventilation and air conditioning; LIS, laboratory information system; QC, quality control.

In the post‐analytical phase, both Abbott and Beckman systems exhibited high‐risk issues of inconsistent test results with clinical features, particularly for CA199 and creatinine assays. These discrepancies were frequently related to heterophilic antibodies or drug‐related interference (Table 5).

**TABLE 5:** Risk assessment of post‐analytical phases in three biochemical and immunological automation systems.

Failure mode	Potential cause	Control measures	ISO 15189:2022 clause	Roche (annual samples: 121,212)				Abbott (annual samples: 490,925)				Beckman (annual samples: 829,645)			
				*S*	*O* (frequency, annual occurrence)	*D*	Risk level	*S*	*O* (frequency, annual occurrence)	*D*	Risk level	*S*	*O* (frequency, annual occurrence)	*D*	Risk level
Reference interval not applicable	Reference interval not sufficiently verified	1. Periodic verification of biological reference intervals	7.3.5	4	2	4	Medium	4	2	4	Medium	4	2	3	Low
					(Once a year, 1)				(Once a year, 1)				(Once a year, 1)		
Result review error	Threshold too wide	1. Periodic verification of automatic review rules	7.4.1.2	4	2	3	Low	4	2	4	Medium	4	2	4	Medium
	Personnel oversight	2. Optimize LIS access control			(Once a year, 1)				(Once a year, 1)				(Once a year, 1)		
		3. Strengthen personnel training													
Delayed critical result reports	LIS/HIS system failure	1. Set up pop‐up reminders	7.4.1.3	N/A	N/A	N/A	N/A	5	2	1	Low	5	3	1	Low
	Personnel response delayed	2. Highlight patient information in yellow							(1%, 143 cases, 1.4)				(1%, 903 cases, 9)		
		3. Strengthen personnel training													
		4. Periodic maintenance of system													
Improper sample storage	High‐risk samples not centrally processed; improper storage temperature	1. Assign personnel to handle high‐risk samples	6.3	3	1	3	Low	4	1	3	Low	3	2	3	Low
		2. Use cold storage with temperature‐and‐humidity–monitoring and alarms			(Once every more than 5 years, 0.2)				(Once every more than 5 years, 0.2)				(Once a year, 1)		
Result‐ clinical inconsistency	Results interfered by heterophile antibodies or drugs	1. Compared with historical results	7.4.1.2	3	2	3	Low	4	5	3	High	4	5	4	High
		2. Consider other related test results			(Once a year, 1)				(Weekly, 52)				(Weekly, 52)		
		3. Consider data from other clinical departments													
Missing items in report	Insufficient sample volume	1. Strengthen training for sample collection personnel	7.4.1.2	4	3	3	Medium	4	2	3	Low	4	2	3	Low
	Clotted samples	2. Periodic maintenance of the classification and cup‐distribution module (R)			(Quarterly, 4)				(Once a year, 1)				(Once a year, 1)		
	Instrument failure	3. Double‐check items during report review													
	Barcode loss/blurring														
	Unclear responsibility distribution														

Abbreviations: HIS, hospital information system; LIS, laboratory information system.

### 3.2 Control Measures for High‐Risk Nodes

To address pre‐analytical sample transport delay, Abbott and Beckman systems introduced the pneumatic tube system (PTS) to achieve immediate sample transport after collection. This intervention significantly improved transport efficiency, reduced in‐transit delay, and ensured workflow continuity. The Roche system, which handles a smaller sample size and includes items with lower time sensitivity, did not adopt the PTS. Instead, its manual transport workflow was optimized to reduce the risk of sample delay. Additionally, all three systems were equipped with data visualization dashboards for real‐time monitoring of sample transport status.

During the analytical phase, Beckman‐specific quality control failures were effectively mitigated through the implementation of the Patient‐Based Real‐Time Quality Control (PBRTQC) system, which continuously monitored patient results to detect analytical drifts and potential biases in real time. Furthermore, reaction curve analysis software was employed to identify abnormal signal fluctuations and provide early warnings, thereby enhancing quality control management under open‐reagent conditions. All three systems were equipped with data visualization dashboards for real‐time equipment monitoring, which reduced failure response time. The Beckman system was further equipped with tablet‐based remote control terminals, enabling real‐time intervention during equipment anomalies to improve response efficiency. To mitigate software failures, a standardized contingency plan was developed and validated through regular personnel training and emergency drills, ensuring rapid recovery during data transmission interruptions. The Beckman system also featured a dual‐backup mechanism, substantially enhancing fault tolerance and operational continuity. To prevent reagent degradation or stock‐out interruptions, all three systems adopted an intelligent reagent management platform and smart refrigerators, enabling real‐time monitoring of reagent temperature and expiration date. This approach reduced manual workload and prevented testing delays caused by reagent shortages.

In the post‐analytical phase, to resolve the discrepancy between test results and clinical features, the Abbott system implemented a multistep verification process for CA199 assays, including automatic dilution rechecks, cross‐validation using the Roche platform and polyethylene glycol (PEG) precipitation, significantly improving result accuracy. For creatinine testing, the Beckman system's middleware automatically performed multi‐rule screening to flag potentially interfered samples, followed by automated dilution and confirmatory testing using the picric acid method, effectively minimizing the risk of reporting errors.

### 3.3 Reassessment After Implementation of Improvement Measures

After intervention, RPN values of high‐risk nodes decreased across all three systems (Table 6). However, the extent of improvement and its contributing factors differed among platforms. Beckman demonstrated a pronounced reduction in quality control–related risks following the implementation of PBRTQC and reaction curve monitoring. Meanwhile, improvements in result‐clinical consistency were more evident in Beckman and Abbott systems, largely attributable to optimized verification strategies and workflow refinement, whereas the Roche system exhibited more incremental but consistent gains primarily associated with workflow coordination and overall system stability.

**TABLE 6:** Risk reassessment after targeted interventions.

Failure mode	Control measures	Roche (annual samples: 129,218)				Abbott (annual samples: 520,125)				Beckman (annual samples: 886,220)			
		*S*	*O* (frequency, annual occurrence)	*D*	Risk level	*S*	*O* (frequency, annual occurrence)	*D*	Risk level	*S*	*O* (frequency, annual occurrence)	*D*	Risk level
Sample transport delay	1. Introduce PTS (A/B)	4	5	1	Low	3	5	1	Low	4	5	1	Low
	2. Data visualization dashboards for real‐time sample status alert		(5.7%, 7365)				(2.3%, 11,963)				(2.1%, 18,611)		
QC failure	1. Introduce PBRTQC system	N/A	N/A	N/A	N/A	N/A	N/A	N/A	N/A	4	5	2	Medium
	2. Real‐time monitoring of reaction curve										(1.45%, 104 items, 550)		
Hardware failure	1. Data visualization dashboards for real‐time instrument status	4	4	1	Low	4	5	1	Low	5	5	1	Medium
	2. Tablet‐based remote viewing and control (B)		(Once a month, 12)				(Once a week, 52)				(Once a week, 52)		
Software failure	1. Establish contingency plan and regular drills	5	1	3	Low	5	2	3	Medium	5	1	3	Low
	2. Real‐time network monitoring												
	3. Dual‐machine backup (B)												
Reagent expiration	1. Smart reagent management platform real‐time alert	4	2	3	Low	4	2	3	Low	5	2	3	Medium
	2. Smart refrigerators		(Once a year, 1)				(Once a year, 1)				(Once a year, 1)		
Result‐clinical inconsistency	1. Specific retesting protocol. For CA199: if the result is high, instrument auto‐dilutes 10×; if deviation > 30%, retest with Roche instrument; if Roche is negative, proceed with PEG precipitation (A)	N/A	N/A	N/A	N/A	4	5	2	Medium	4	5	2	Medium
	2. Creatinine multi‐rule analysis applied to assess drug interference (B)						(Once a week, 52)				(Once a week, 52)		
	3. Increase the picric acid method for creatinine retest (B)												

Abbreviations: PBRTQC, Patient‐Based Real‐Time Quality Control; PTS, pneumatic tube systems.

## 4 Discussion

This study, based on the ISO 15189:2022 framework, adopted an integrated qualitative–quantitative FMEA approach to systematically assess risks across the pre‐analytical, analytical, and post‐analytical phases of multibrand biochemical and immunological automation systems at Peking University Shenzhen Hospital. The findings revealed that the risk types and corresponding improvement measures in TLA systems differ substantially from those in conventional laboratory workflows. While Roche, Abbott, and Beckman platforms shared several common high‐risk nodes, they also exhibited distinct system‐specific risk characteristics. By embedding ISO 15189:2022 requirements into the entire risk management process, the study established a standardized path for risk identification, control, and continuous improvement, achieving cross‐system comparison and optimized management among multiple automation brands.

From a methodological perspective, the study incorporated key ISO 15189:2022 clauses (5.6, risk management, 6.3, facilities and environmental conditions, 6.4, equipment, and 7 process requirements) into the evaluation framework. This ensured full coverage of potential failure modes and their impacts on patient safety throughout the entire testing process, thereby minimizing blind spots and promoting continuous quality improvement. Integration of these standards not only enhanced the consistency of risk assessment but also reinforced attention to critical factors such as equipment validation and environmental control.

Risk identification and assessment constitute the core of the risk management cycle, with the occurrence score being a crucial determinant. This study broke through traditional FMEA's reliance on qualitative or semiquantitative scoring by combining quantitative data with expert‐based qualitative evaluation. Actual frequency data from LIS, equipment logs, and quality control software were integrated with expert consensus scoring and standardized to a five‐point scale. This multidimensional approach improved objectivity, comparability, and reproducibility, enabling a more rigorous evaluation of complex risk scenarios and providing a replicable model for risk management in automated laboratory systems.

In 2024, the Roche, Abbott, and Beckman systems processed 129,218, 520,125, and 886,220 samples, respectively, encompassing processes such as sample transport, module coordination, data transmission, and result verification. Compared with conventional laboratory modes, these high‐throughput systems exhibited greater workflow complexity and higher risk exposure. Hardware malfunctions could trigger cascading failures across interconnected modules, while LIS interruptions could cause widespread data‐chain disruption. Additionally, centralized high‐throughput processing amplified the impact of isolated errors and largely unmanned continuous operation might create surveillance blind spots [13]. Pre‐analytical errors, such as barcode errors, transport delay, and sample confusion, may propagate downstream and become major causes of reporting delay [14, 15]. Similarly, post‐analytical issues such as result‐clinical inconsistency could delay clinical decision‐making, underscoring the need for dynamic, system‐wide risk control. Accordingly, this study developed a full‐process risk assessment framework with intelligent targeted interventions to suppress potential failures, thereby enhancing both quality assurance and patient safety.

In line with ISO 15189:2022 clause 5.6, multiple intelligent control strategies were integrated to address the identified high‐risk nodes. During the pre‐analytical phase, PTS were introduced in the Abbott and Beckman platforms to expedite sample delivery and reduce in‐transit delay, while all three systems implemented data visualization dashboards for real‐time monitoring of sample status. In the analytical phase, standardized contingency plans, dual‐backup mechanisms, and regular emergency drills were established to mitigate equipment failures and data transmission interruptions. Beckman's system‐specific quality control instability was effectively resolved through implementation of PBRTQC coupled with real‐time reaction curve monitoring, enabling early detection of analytical drift and deviation [16, 17]. Furthermore, all three systems adopted smart reagent management platforms and smart refrigerators for continuous monitoring of lot numbers, expiration dates, and storage conditions, thereby preventing reagent‐related interruptions. In the post‐analytical phase, intelligent verification algorithms were used to flag potentially erroneous results for secondary review, significantly improving reporting accuracy. Overall, the RPN values of key failure nodes markedly decreased after intervention, demonstrating the superior efficiency of intelligent control compared with conventional manual management.

The observed differences in risk profiles and intervention effectiveness among the three automation systems can be largely attributed to their distinct technical architectures and operational characteristics. Beckman's platform, which partially employs open reagents, was inherently more susceptible to quality control‐related instability prior to intervention, rendering it particularly responsive to PBRTQC and reaction curve–based surveillance. In contrast, the use of closed reagent systems in Roche and Abbott platforms shifted the predominant risk burden away from analytical instability and toward workflow coordination, data transmission, and post‐analytical processes. Accordingly, verification‐oriented strategies and process optimization resulted in more pronounced improvements in Beckman and Abbott systems, particularly with respect to result‐clinical consistency. These findings highlight the necessity of tailoring risk control measures to system‐specific vulnerabilities rather than adopting a one‐size‐fits‐all approach.

Most previous studies focused on risks associated with single instrument or isolated process, rarely encompassing the entire process from pre‐analytical to post‐analytical phases or performing multibrand comparisons within the same institution [18, 19, 20]. In contrast, this study conducted simultaneous cross‐brand risk identification and evaluation across Roche, Abbott, and Beckman systems under the same operational environment, thereby revealing both shared and differential risks in high‐throughput automated settings. This work addresses an important research gap by integrating multisystem comparison with ISO 15189:2022 compliance mapping.

Several limitations should be acknowledged. First, this study was conducted in a single institution and involved three specific automation pipelines that differ in system configuration and assay scope. Specifically, the Beckman system performs both biochemical (partly using open reagents, which is common practice in China) and immunological testing, whereas the Roche and Abbott systems primarily handle immunoassays. Therefore, the generalizability of these findings may be limited. Laboratory managers should tailor risk control strategies according to system characteristics, including workload, automation level, and key vulnerability. For high‐throughput systems, priority should be given to improving transport efficiency and implementing anomaly alerts, while platforms with strong information dependency may require greater emphasis on interface stability and data integrity. Furthermore, establishing dynamic, data‐driven risk‐monitoring mechanisms with real‐time updates to intervention strategies may further reduce risk and optimize resource allocation [21, 22]. With the increasing integration of automation and artificial intelligence (AI), future research should explore AI‐driven adaptive risk recognition and early‐warning frameworks to advance laboratories toward intelligent and data‐centric quality management [23].

## 5 Conclusion

Based on the ISO 15189:2022 standard and the FMEA methodology, this study systematically evaluated and controlled full‐process risks across three biochemical and immunological TLA systems at Peking University Shenzhen Hospital, achieving cross‐brand comparison and optimized management. By integrating quantitative data with expert judgment, a scientifically robust hybrid scoring system was established to support targeted improvement measures. Following implementation of intelligent control strategies, high‐risk nodes were effectively mitigated, resulting in improved analytical reliability, operational stability, and patient safety. This work provides both theoretical foundations and practical guidance for risk management in high‐throughput automated laboratories and lays a foundation for future intelligent quality management systems.

## Author Contributions

M.W. was involved in data analysis and writing. M.L. was involved in collecting data and editing. Y.L. helped in collecting data. B.L. performed data analysis. H.X. was involved in data analysis, writing – review, and revision. Y.X. performed study design and revision. All authors reviewed the manuscript.

## Conflicts of Interest

The authors declare no conflicts of interest.

## Data Availability

The data that support the findings of this study are available from the corresponding author upon reasonable request.
